# On Serendipitous Campus Meetings: A User Survey

**DOI:** 10.3390/ijerph192114504

**Published:** 2022-11-04

**Authors:** Sascha Naomi Jansz, Mark Mobach, Terry van Dijk, Esther de Vries, Roeland van Hout

**Affiliations:** 1 Department of Spatial Planning and Environment, Faculty of Spatial Sciences, Rijksuniversiteit Groningen, 9712 CP Groningen, The Netherlands; 2 Research Group Facility Management, Research Centre for Built Environment—NoorderRuimte, Hanze University of Applied Sciences, 9747 AS Groningen, The Netherlands; 3 Research Group Spatial Environment and the User, The Hague University of Applied Sciences, 2521 EN The Hague, The Netherlands; 4 Tranzo, Tilburg School for Social and Behavioral Sciences, Tilburg University, 5000 LE Tilburg, The Netherlands; 5 Centre for Language Studies, Radboud University, 6500 HD Nijmegen, The Netherlands

**Keywords:** campus design, spaces, services, serendipity, unplanned meetings, interactions

## Abstract

With campuses opening up and stimulating interactions among different campus users more and more, we aim to identify the characteristics of successful meeting places (locations) on campus. These can help practitioners such as campus managers and directors to further optimize their campus to facilitate unplanned or serendipitous meetings between academic staff and companies. A survey on three Dutch campuses, including questions on both services and locations, was analyzed both spatially and statistically using principal component (PC) and regression analysis. Four PCs were found for services (Relax, Network, Proximity and Availability) and three PCs were found for locations (Aesthetics, Cleaned and Indoor Environment). Personal characteristics as explanatory variables were not significant or only had very small effect sizes, indicating that a campus’ design does not need to be tailored to certain user groups but can be effective for all. The pattern of successful locations is discussed, including the variables in each PC. These PCs provide a framework for practitioners who want to improve their campus’ design to further facilitate unplanned meetings, thus contributing to cooperation between campus users, hopefully leading to further innovation.

## 1. Introduction

The three main objectives of Dutch universities are education, research and knowledge valorization [1]. As described by Etzkowitz [2], knowledge valorization is often stimulated by a triple helix of academic–industry–government relationships. One of the things that sets aside a university campus from a generic collection of buildings is that many universities seek to create meeting places for all actors, in part also by attracting more companies to their campuses [3,4,5]. These meeting places are expected to foster unplanned meetings among all different campus users, as these may lead to collaboration and knowledge sharing [6]. As such, these unplanned meetings are a significant step towards innovation and considered to be essential to achieving university valorization goals [7,8].

Many campus directors seek to further optimize their campuses to facilitate these unplanned meetings, but providing the required attractive and versatile range of accommodations and facilities constitutes a struggle [3,9]. In this paper, an unplanned meeting will be defined as a serendipitous meeting between at least two people that is a direct result of coincidental visual contact. To specify further, following Brown [10] (cited by [11] p. 878), intentional unscheduled visits (the initiator of the conversation walked up to someone) are not included. The focus is on conversations initiated after coincidental visual contact (partners happened to see each other and started talking). Unplanned meetings at (networking) events are included, as the participants at these events do not know in advance which other participants will be there and/or if there will be an opportunity to speak to a specific person. This study does not include digital meetings. Serendipity has been described by van Andel [12] as “the art of the unsought finding”. The serendipitous communication model, as described by Peponis [13], suggests that informal interaction nodes such as coffee machines, hallways, seats, cafés and other facilities help to bring people together outside of normal workspaces, as they encourage frequent unplanned interactions [11,13] and stimulate so-called weak ties between people [14]. This paper looks at these informal interaction nodes from two angles: locations (or spaces) (hallways, seats, etc.) and services (facilities such as coffee machines, cafés, etc.).

As described by Björneborn [15], serendipity cannot be engineered or designed per se, but we can design the affordances for serendipity; in this case, for unplanned meetings. This is as seen from the designers’ point of view. From the users’ point of view, serendipity must always be encountered in unplanned ways in order to be serendipitous [15] (p. 23). In other words, unplanned meetings as used in this study are unplanned by the users themselves but may be partly induced by the campus designers. The theory of affordances as described by Gibson states that we do not perceive the qualities of an object or environment, but the affordances it offers us. As such, to humans, a handle affords grasping [16,17]. Furthermore, Fayard and Weeks found that these affordances are related to privacy (people must be able to control the boundaries of their conversation), propinquity (people must come into unplanned contact with others) and social designation (people must feel it is socially acceptable to stop and talk to each other). The elements that had a positive or negative effect on privacy and propinquity in their study were architectural elements, geographical elements and functional elements [16]. This means that the built environment can affect the unplanned meetings taking place, as settings can vary in the extent to which these afford informal interactions. Some settings will foster these types of interaction, while others may not [16].

To help campus managers identify which affordances can stimulate unplanned meetings between academic staff and company employees and how these can be successfully implemented in campus design, this paper aims to investigate the following research questions:

(1) In which locations on campus do these serendipitous or unplanned meetings typically take place?
(2) What are the service and spatial characteristics of these successful locations?
(3) What design elements in campus spaces and services could be providing the necessary affordances for serendipitous or unplanned meetings?

To the best of our knowledge, no study has yet looked at a multitude of successful unplanned meeting locations across Dutch university campuses. This may be because it is hard to identify these locations. Even though campus directors can identify locations where they expect these meetings to take place, or on which they have had a lot of positive feedback [18], they have no way of knowing how much better one location is over the other, let alone why these differences occur. Therefore, the user’s perspective is needed: which locations do campus users indicate they have had successful unplanned meetings in?

This paper aims to investigate the locations reported by users where unplanned meetings between academics and company employees took place on three Dutch university campuses, to identify what facilities contributed to these meetings, and if these have a common set of general spatial characteristics.

To be able to quantify spatial characteristics, Mehrabian and Russel developed a list of environmental descriptors. They proposed that environmental stimuli are linked to the behavioral responses of pleasure, arousal and dominance, three independent emotional dimensions to describe a state of feeling [19,20]. They concluded that a person’s environment directly affects their behavior. Respondents were asked to score a number of variables on an evaluative continuum. E.g., for pleasure they used adjectives such as happy–unhappy, for arousal, excited–calm, and for dominance, controlling–controlled [20]. These dimensions were uncovered through a principal component analysis (PCA), based on a list of opposite evaluative adjectives such as pleasant–unpleasant and colorful–drab, where a respondent would score on descriptors such as pleasant, bright, colorful, organized, ventilated, elegant, impressive, large, modern and functional [19].

In a previous study by the authors of this paper [18], the facility directors of 13 Dutch university campuses were asked what they felt were critical success factors that influence interaction on campuses. These respondents reported that a campus should be seen as an open system where everything is affected by its context. Within this context, six abstract categories that influence interaction were derived from the data: constraints, motivators, designing spaces, designing services, building community and creating coherence. These were further specified in variables such as coffee, meeting spaces, shared research facilities, event programming and networks [18].

This paper takes the view of the campus users and seeks to identify and explain concrete locations where unplanned successful meetings took place.

## 2. Materials and Methods

### 2.1. Study Population

The study population consisted of the campus users of 3 Dutch university campuses. As this study aims to identify locations of unplanned meetings between campus users from companies and knowledge institutions, the top 3 campuses with the most companies, as defined in a study by Buck [21], were selected. These were “Kennispark Twente” (UT—471 companies), “TU Delft Science Park” (TUD—245 companies) and “Campus Groningen” (RUG—198 companies). On these campuses, both university and company employees were invited to participate in the survey. The response from company employees was too low to be representative and has therefore been excluded. Moreover, university support staff were excluded as they are unlikely to be looking for cooperation and knowledge sharing with on-campus companies. The target group for the survey, therefore, was the academic staff, with a study population (N) on the 3 campuses as follows: UT (1907); TUD (2750); RUG (2012).

### 2.2. Data Collection

The recruitment of participants in this study was facilitated by the respective boards of the 3 universities granting permission to send out invitations to relevant university email addresses. (Online participation was required due to COVID-19 “stay at home” measures at the time.) Permission was granted to invite 100% of academic staff at UT (1907 invites) and RUG (2012 invites). TUD granted permission to invite 14% of academic staff (393 invites). In the first week of September 2020, respondents received an email invitation to participate in the study through the internet-based survey engine Maptionnaire [22]. A first reminder was sent on 14 September, a final reminder was sent on 28 September, indicating that the survey would remain open until 30 September 2020.

After obtaining informed consent of all participants, the framework of the study was introduced, and important definitions were explained (e.g., partners: contacts from companies (business partners) or knowledge institutions that are or could be cooperation partners and can share knowledge important to innovation and valorization in your work). Then, respondents were asked to indicate their base location on the map (the place where they generally work on campus), as well as “the most important location on campus where you have met people from companies who later became partners”, followed by “the most important location on campus where you have met people from knowledge institutions who later became partners”. The survey was available in both English and Dutch.

After indicating a location on the map, a pop-up followed asking to rate that location on a 7-point Likert scale for 20 spatial characteristics, based on the environmental descriptors as defined by Mehrabian and Russell [19]. Finally, respondents were also asked to indicate what 13 service variables contributed to these interactions, again on a 7-point Likert scale and based on the previous study mentioned above [18] (see Section 2.6). Participants were asked to answer all questions based on their experiences in 2019, to ensure the COVID-19 measures in place during the study would not influence the results.

### 2.3. Campus Description

The campuses included in this study are university campuses, meaning that the university is the main campus user. Table 1 contains information about their characteristics.

**Figure 1:**
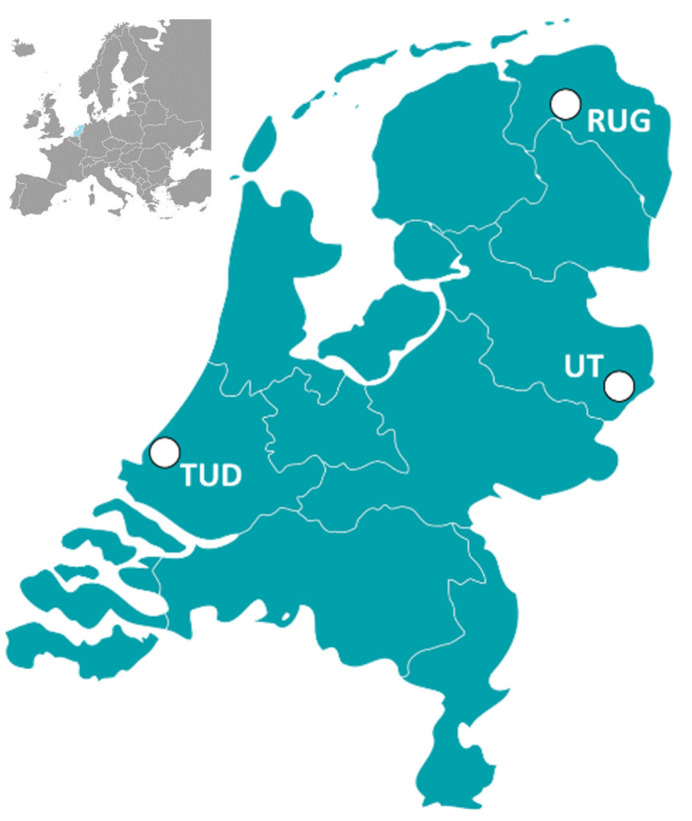
Locations of the three studied university campuses in The Netherlands.

In this paper the term “campus” is used as defined by Dinteren, Jansen and Lettink [9]: “an active open innovation environment where actors can meet and inspire each other in the presence of high-end shared facilities and at least one renowned knowledge carrier, which largely determines the campus’ thematic profiling” (in this case the university). Therefore, it does not only include the university’s buildings, but also those of other knowledge institutions and/or companies on the same site. Figure 1 shows the locations of the three studied university campuses in The Netherlands.

### 2.4. Volunteered Geographic Information (VGI) and Public Participation Geographic Information System (PPGIS)

Data was collected using volunteered geographic information (VGI), a form of a public participation geographic information system (PPGIS) combining traditional surveys with online maps [26,27]. As demonstrated by Goodchild [27], and Goodchild and Li [28], VGI can be used to capture and map perceptions and objective properties of places. For example, Soares, Weitkamp and Yamu [29] used VGI to explore perceptions and experiences of creative encounters at university campuses and science parks on 2 campuses in The Netherlands.

**Figure A1:**
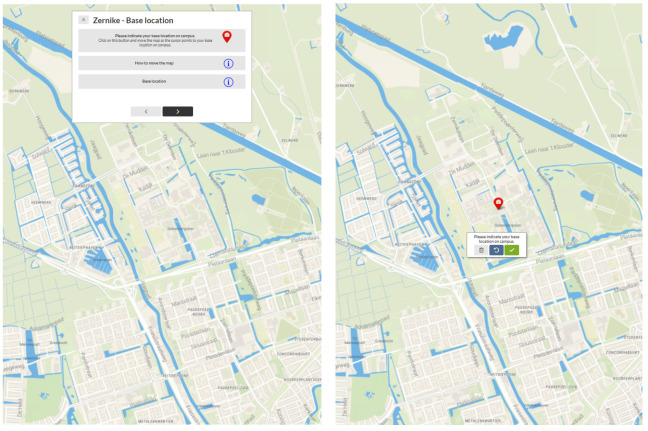
RUG campus—Maptionnaire screenshot: indicating base location.

**Figure A2:**
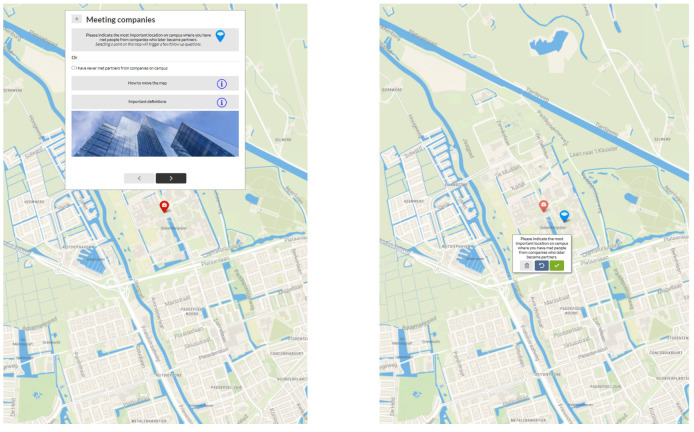
RUG campus—Maptionnaire screenshot: indicating meeting location.

This study used Maptionnaire [22] to record unplanned meeting locations and collect perceptual data about these locations. Screenshots are provided in the Appendix A (see Figure A1 and Figure A2). Although socio-demographic data of the respondents was collected, the focus of this study is on the indicated locations and related spatial and services perceptions of the respondents.

### 2.5. Data Preparation and Analysis

**Figure A3:**
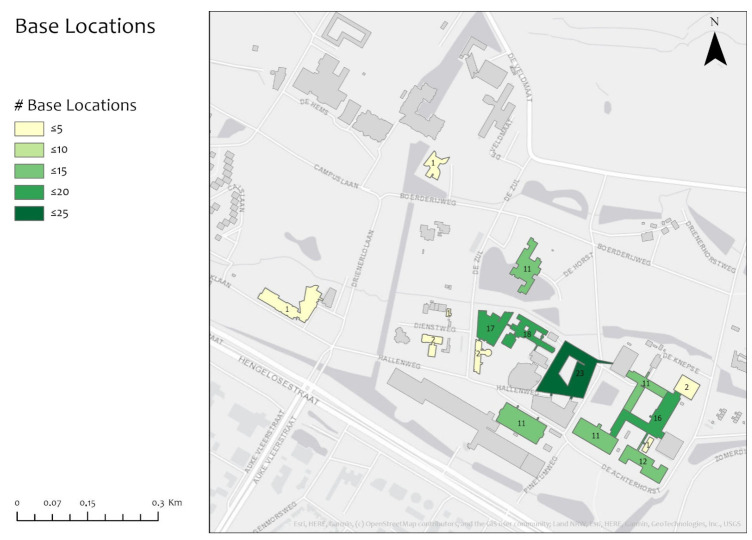
UT campus—number of base locations. # = ‘Number of’.

**Figure A4:**
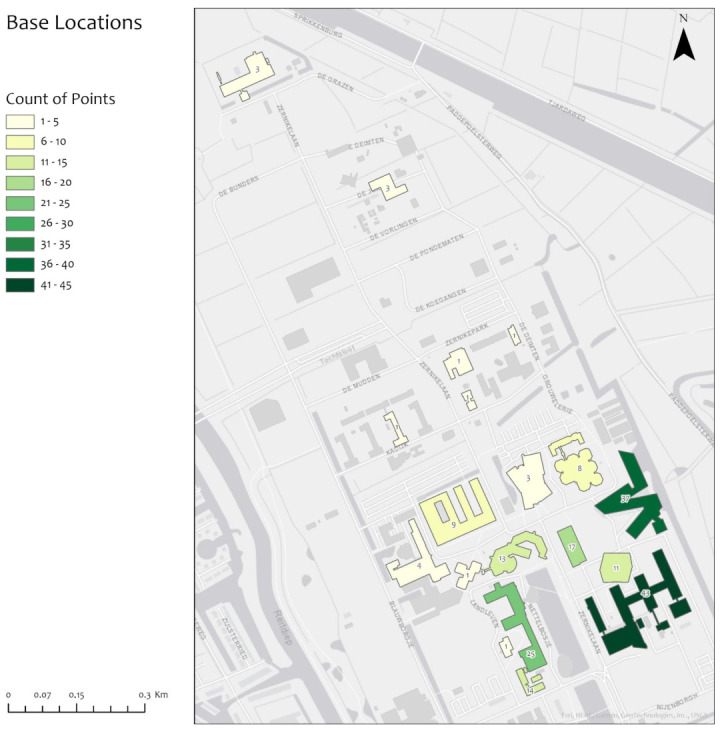
RUG campus—number of base locations.

**Figure A5:**
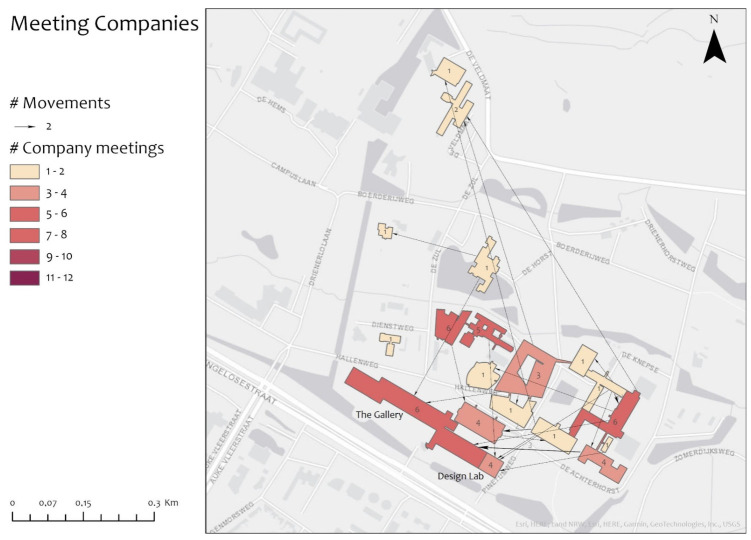
UT campus—meetings with companies and movement across campus. # = ‘Number of’.

**Figure A6:**
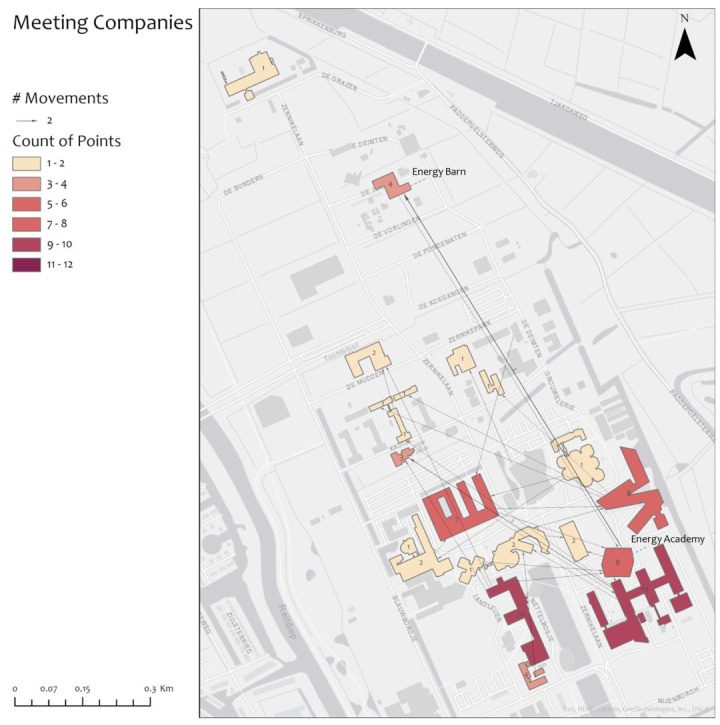
RUG campus—meetings with companies and movement across campus. # = ‘Number of’.

**Figure A7:**
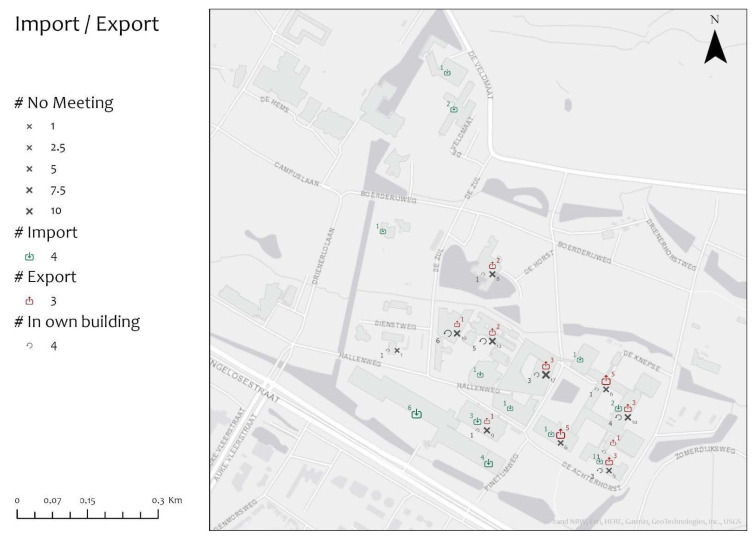
UT campus—import/export of unplanned meetings with companies. # = ‘Number of’.

**Figure A8:**
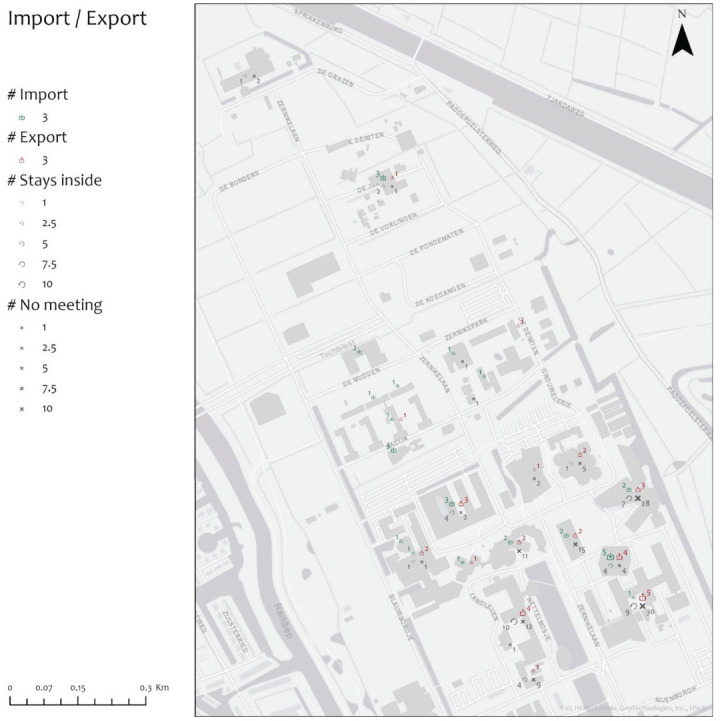
RUG campus—import/export of unplanned meetings with companies. # = ‘Number of’.

**Figure A9:**
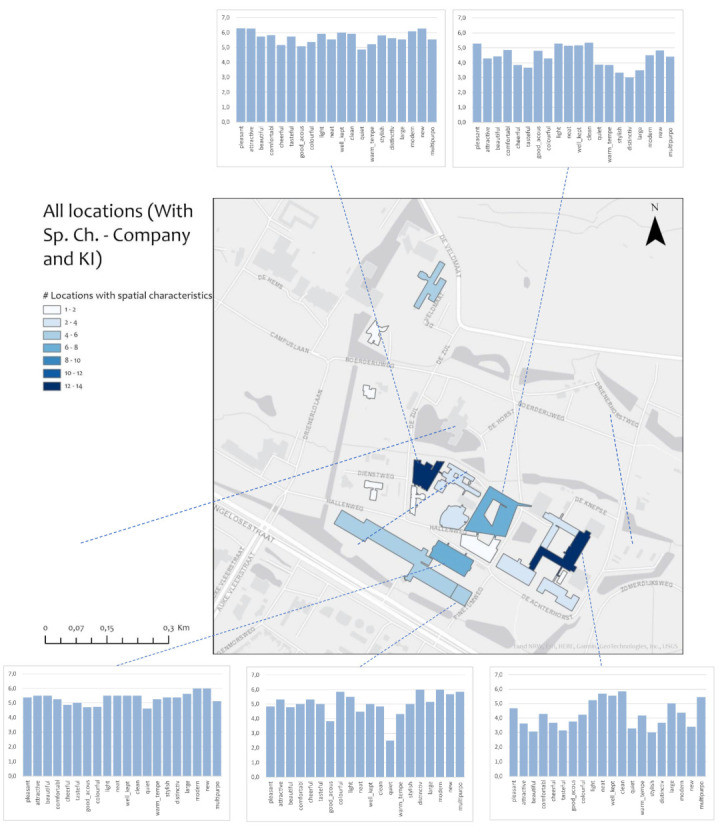
UT campus—average building characteristics’ score for buildings with the highest number of indicated locations. # = ‘Number of’.

**Figure A10:**
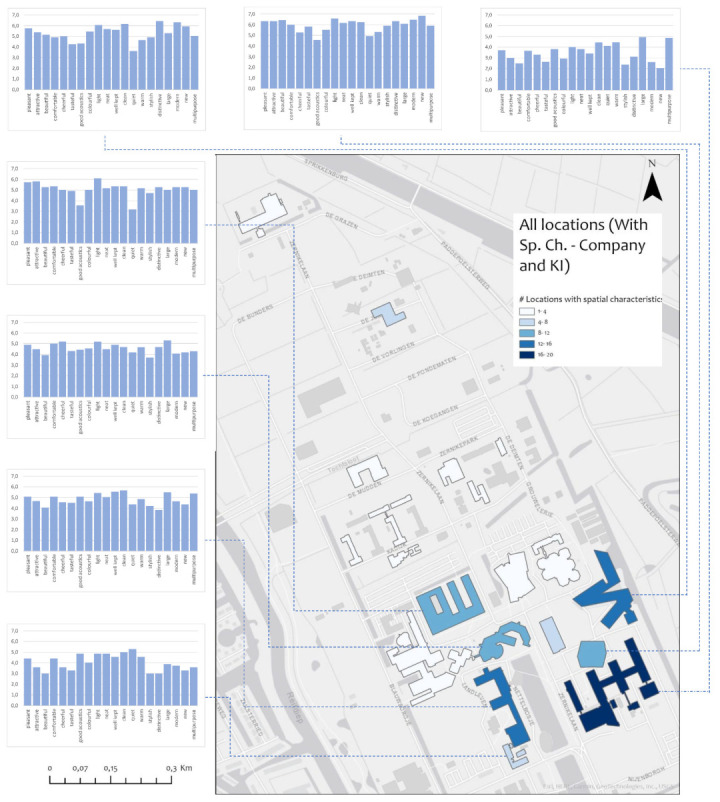
RUG campus—average building characteristics’ score for buildings with the highest number of indicated locations. # = ‘Number of’.

The collected data shows the locations on campus where university academic staff (the respondents) have indicated they had met people from companies who later became partners. Survey data was downloaded from Maptionnaire into ArcGIS [30]. The data was then reviewed, removing points outside of the campus boundary or in impossible locations (e.g., in a body of water). Points within 10 m of a building were assigned to that building. If a point was within the 10 m range of multiple buildings, it was assigned to the closest building. Points that were not within 10 m of a building were allocated to “outside”. Then, the following summative maps were rendered showing: (1) the number of base locations (the place where you generally work on campus) per building; (2) for each building, the number of respondents who indicated they had met later work partners there; (3) where respondents came from to have these meetings; (4) how many respondents in each building indicated either: no meeting at all; a meeting in this building and the respondent was based here; or a meeting in this building but the respondent came from a different building and had left the building to have a meeting elsewhere; and (5) how respondents rate the environmental descriptors of the location on the 7-point scale for spatial characteristics (see Figure A3, Figure A4, Figure A5, Figure A6, Figure A7, Figure A8, Figure A9 and Figure A10).

While the maps show only the locations where respondents met with people from companies, the statistical analyses also use the data for meetings respondents had with knowledge institutions. As an unplanned meeting is unplanned, respondents would not know in advance if they were meeting with someone from a company or from a knowledge institution. We therefore assumed that there were no differences in how respondents would rate the service and spatial characteristics, whether they met someone from a company or met someone from a knowledge institution. Moreover, to find a general (not campus-specific) pattern of success factors, the statistical analyses include the data from all 3 campuses.

The dataset was split in 2 parts: 1 containing all indicated unplanned meeting locations and respondents’ perceptions about these locations (location dataset) and 1 containing respondents’ perceptions about services that contributed to these unplanned meetings (services dataset). Both datasets were analyzed in 2 steps. First, a PCA was performed to reduce the number of variables and identify more general components. Second, a linear (mixed) regression was performed to find patterns in the data and to investigate explanatory variables.

### 2.6. Variables

For location and service variables, all variables were asked on a 7-point Likert scale, ranging from 1 (strongly disagree) to 7 (strongly agree) (see Table 2). These variables were selected based on Mehrabian and Russell [19] (for location variables) and a previous study by these authors [18] in which university facility directors identified key success factors for unplanned meetings (for service variables).

As the location and services variables are based on a 7-point Likert scale, ranging from 1 (strongly disagree) to 7 (strongly agree), these can be interpreted as ordinal variables. The order in the ordinal-level data is clear, but the absolute distance between the levels is unknown [31] (p. 1). Agresti explains that there are 2 ways to analyze ordered categorical response variables: (1) to ignore the categorical nature and use “standard parametric methods for continuous response variables”, such as linear regression, or (2) to restrict analysis “solely to methods that use only the ordering information about the categories” [31] (p. 3). However, as described by Tabachnick and Fidell [32] (p. 7), these variables are often treated as if they are continuous in practice, because the underlying scale is thought to be continuous. This is also assumed to be the case in this study.

The collected personalia variables were provided by the respondents themselves (see Table 3). A total of 2 variables were added to the data by the authors: “quality” and “campus” (see Table 4). The variable “quality” was assigned to indicate if a building had been designed with (unplanned) meeting in mind. This was done based on building descriptions on the university/campus website and conversations with campus managers. Nevertheless, this variable is only an indication, as this was not assigned by the designers of these buildings themselves (unless stated in the building description, with was often the case for newer buildings, but not for older ones). The variable “campus” was available because of the survey design.

### 2.7. Principal Component Analysis (PCA)

As described by Joliffe [33], PCA is a verified and reliable technique to reduce a large number of interrelated variables into a smaller set of components while retaining the variation in the dataset as much as possible. ”This is achieved by transforming existing variables into a new set of variables, the principal components (PCs), which are uncorrelated, and which are ordered so that the first few retain most of the variation present in all of the original variables” [33] (p. 1). As such, PCA has been used to reduce the number of variables in spatial data before, for examples see [34,35].

To reduce the number of variables and to trace more general PCs, a PCA was performed on each dataset (locations as well as services). To test for possible biases, the PCA was performed on different selections of the dataset: on the original dataset, per campus, including missing values, replacing missing values with series mean and filtered for more than two missing values. Only factors with eigenvalues above 1.0 were retained [32]. The results were consistent and showed that the original dataset (including missing values and excluding cases pairwise) could be used for the PCA.

The pattern of factor loadings is interpreted by the authors to determine their meaning and to assign an interpretive label to the newly derived PCs [36]. After analyzing the resulting factor loadings, we chose the minimum threshold of 30% overlapping variance, or 0.55, which is described by Tabachnick and Fidell [32] as good. The resulting factor loadings can be seen in Section 3.3.3 and Section 3.4.3. The outcomes of the Kaiser–Meyer–Olkin (KMO) score and the Bartlett test of sphericity are shown in Table 5 (services) and Table 6 (locations). For services: with a KMO of 0.747, well above 0.5, and a Bartlett test significance of 0.000, well below 0.05, the PCA was continued [37]. For locations: with a KMO of 0.931, well above 0.5, and a Bartlett test significance of 0.000, well below 0.05, the PCA was continued as well [37].

As explained in Section 3, the PCA resulted in 4 PCs for services (Relax, Network, Proximity and Availability) and three PCs for locations (Aesthetics, Cleaned and Indoor Environment).

### 2.8. Regression Analysis

For the regression analyses, the newly constructed PC scores were used as the dependent variables. These PC scores are based on the location and services variables described above, with the assumption that the underlying scale is continuous. They can therefore be used as dependent variables in regression analyses [32] (p. 7).

For services, a common linear regression model (LM: lm package in R) was used. It was tested if personal characteristics such as age, worktime and role were relevant variables in explaining the PC scores. To evaluate the possible regression models, we used Akaike’s information criterion (AIC) values.

For locations, a linear mixed effects regression (LMER: lmer package in R) was used, because the variance in these data could not only arise from differences between participants (like for services), but also from differences between locations (where meetings took place) and between base locations (the place where participants generally work on campus). This means that there are three potential random variables: participants, locations and base locations. Evaluating the possible regression models, using AIC values, it turned out that participants and locations were meaningful random variables. Adding “base location” as a third random variable did not improve the regression model. Therefore, the LMER analyses were restricted to participant and location as random variables; we used their random intercepts in the analyses. We also tested random slopes between these two random variables and the explanatory variables, but these interactions had no significant impact (again tested by using AIC values).

## 3. Results

### 3.1. The Sample

**Figure 2:**
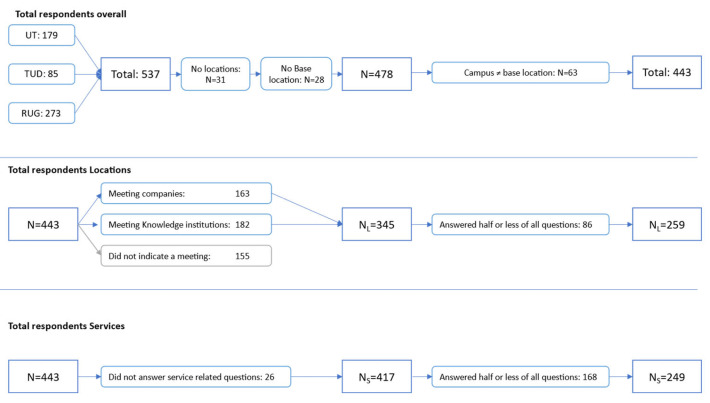
Flowchart cleaning datapoints.

There were 537 respondents in total, or a 12.5% response rate overall, divided as follows: UT, 179 responses, 9.4% response rate; TUD, 85 responses, 22.4% response rate (3.1% of total study population); RUG, 246 responses, 12.2% response rate (all including both partially and fully completed surveys). Respondents who did not indicate any location were removed (N = 31). In addition, respondents who indicated a meeting location but not their base location were removed (N = 28), as well as those who indicated the campus was not their base location (N = 63), resulting in a total of N = 443 responses (see Figure 2).

Respondents indicated 163 locations for meetings with companies and 182 meetings with knowledge institutions. Some respondents did not indicate any meeting location (N = 155) as they had not had a meeting with people from companies or knowledge institutions who later became partners in 2019. Some respondents indicated more than one meeting location. This resulted in data on 345 meeting locations (N_L_), and data on 417 responses regarding services (N_S_).

In each set, “Don’t know” answers were removed. Finally, respondents answering only half or less of all questions were removed, resulting in a final dataset of N_L_ = 259 and N_S_ = 249. The number of points “outside” (not within 10 m of a building) for each campus were: RUG: N = 4, UT: N = 3, TUD, N = 0.

### 3.2. Spatial Descriptives

Unfortunately, the responses rate on the TUD campus was too low to be representative (only 3.1% of the total study population, versus 9.4% for UT and 12.2% for RUG). This low response rate on the TUD campus was likely because permission was only granted to invite 14% of the population to the survey. TUD will therefore not be included in the spatial descriptives.

Figure A3 and Figure A4 show the concentration of base locations per building. The darker green the color, the more base locations have been indicated. On both campuses it is clear that most respondents are based in the most densely built part, or “campus center”. On both campuses this happens to be the southeast side of the campus. There are also multiple buildings where no base locations were indicated (grey). It is therefore important to keep in mind that these results represent the perceptions of academic staff located in the campus center.

Figure A3 and Figure A4 show the number of indicated unplanned meetings with companies per building; the darker red the color, the more meeting locations have been indicated. The concentration of these meetings is also in the campus center. On both campuses, meetings have been indicated in buildings that were (mostly) occupied by companies, showing that academic staff also enter these buildings occasionally. It is also clear that many respondents did not indicate a meeting. As the number of respondents from companies was too low to be included in these results, it is important to keep in mind that only the travel patterns of academic staff are visible. Company employees may also visit university buildings, but this is not included in the results.

Figure A5 and Figure A6 show the number of indicated meetings with companies per building, and where these respondents came from (their base location). It is clear that there is movement across the campuses. At UT there is a clear movement towards “The Gallery” (six indicated locations, plus four indicated locations for the design lab located in the same building.) “The Gallery” is a modern building intended for high-tech innovation, knowledge valorization, and innovative entrepreneurship [38]. The other three buildings with more than five indicated meetings have not attracted these from other buildings, but were indicated by respondents who had their base location in the same building. At RUG there is a clear link with traffic between the Energy Barn and the Energy Academy (respondents having their base location in one and reporting a meeting in the other). In addition, the company buildings located around the Kadijk have attracted meetings from respondents located elsewhere on the campus. As this is a company location, respondents located on the Kadijk may also have meetings elsewhere on the campus, but these would not have been captured in the data, as there was insufficient response from company employees. Once again, university buildings with the highest number of reported unplanned meetings have mostly captured respondents that stayed within their own building.

Figure A7 and Figure A8 show the same data in a different way: they show whether indicated meetings were mostly from respondents with their base location in the same building, or if those respondents had left their building when they experienced an unplanned meeting. They also show that most respondents did not indicate having an unplanned meeting on campus at all.

The Likert scores respondents gave to their indicated locations, rating them on 20 location characteristics based on Mehrabian and Russell’s [19] environmental descriptors, may give an indication on whether perceived building characteristics can be an explanatory variable for unplanned meetings. Figure A9 and Figure A10, therefore, show the average score of the individual environmental descriptors for the buildings with the highest numbers of indicated meetings on these spatial characteristics. There is not enough data on these spatial characteristics for buildings with a low number of indicated meetings, as respondents only rated their indicated locations.

At UT, the general score of all buildings is positive (an average above 4 out of 7). The two buildings with the highest number of indicated meetings do show differences. Building #10 scores very high overall, but building #20 has more variation across the different characteristics (see Figure A9).

At RUG, it stands out that the building with the highest number of reported unplanned meetings has the lowest score on building quality overall. Therefore, it does not seem that building quality is the main predictor of unplanned meetings.

### 3.3. Services

#### 3.3.1. Respondents

As can be seen in Table 7, 50% of the analyzed responses for services (n_S_ = 249 see Figure 2) are from RUG and the majority of respondents are male. There is representation from all levels of academic staff. On average, respondents are 38 years old and have worked at their campus for seven years. They reported to have met an average of two people from companies who later became partners and two people from knowledge institutions who later became partners in 2019 (see Table 7).

#### 3.3.2. Principal Component Analysis (PCA)

To reduce the number of variables and discover more general components, a PCA was performed. The PCA resulted in four new PCs with an eigenvalue >1, explaining 60.6% of the original variance. Table 8 shows the four PCs resulting from this PCA after varimax rotation.

To determine meanings by assigning appropriate labels to the newly derived PCs, the pattern of factor loadings was interpreted on the basis of the factor loadings in Table 7. The factor loadings in bold make clear that most variables have high loading on only one factor, the exception being the meeting variable that loads on both the second and fourth factor. Two variables do not load high on any of the four factors. We labeled the four PCs (indicated with a capital letter) and their meaning as follows:

PC1, Relax: Food and drinks, such as lunch services and coffee, are important contributors. To have a “nice place to be”, where the food and drinks can be consumed, or a nice area where campus users can take a walk during their (lunch) break are also important. As these variables all relate to user’s activities when they are taking a (short) break from work, this PC has been named Relax.

PC2, Network: Respondents indicated that having a meeting strongly contributed to their interactions with people they had not met before. Additionally, participating in events was important. Both are great opportunities to have a chance introduction by a coworker. As these variables were all related to being able to build their network, this PC has been named Network.

PC3, Proximity: Being close to your base location or being on your daily route across campus are also strong contributors. Shared (research) facilities can also create a need to be in a certain place, apart from your daily route and base location, where you can meet new people. All three relate to not having to go out of your way to have unplanned meetings, which is why this PC has been named Proximity.

PC4, Availability: This PC contains two similar variables: “meeting” and “(meeting) room” are closely connected, but not the same: a meeting implies that a (semi) private location has been arranged, while a (meeting) room refers to having a space available to talk to a new connection you have just made. Both are strong contributors. The authors found this PC harder to name, but as having a meeting is also represented in Network, this PC was assigned the name Availability.

The variable “meeting” has a double loading, both in Network and in Availability. It fits well in both, as a meeting is generally planned, ensuring there is a space available and that there are people there that are relevant to the meeting. Two variables are not included in these PCs: “Printer services” and “There is always something interesting going on here”. This can either mean that these did not contribute to the unplanned meetings, or that they were not relevant in distinguishing the specified locations.

#### 3.3.3. Personal Characteristics as Explanatory Variables

As described in the methodology section, a linear regression model (LM) was used to test which of the seven personal characteristics (see Table 7) has an impact on the PC scores for services. Each characteristic was tested separately; thereafter, we tested all characteristics taken together in one LM without interactions. Finally, we tested all possible combinations of two characteristics (two-way interactions) and selected those combinations of characteristics including interactions which had a significant relation. It turned out that most characteristics and characteristic combinations were not related to the PC scores.

The final best models according to the AIC values are given in Table 9. The outcomes show that only two personal characteristics, Age and Worktime, were significant as explanatory variables for services—not more than maximally one per PC, with small effect sizes. There were no campus differences.

For Relax, there were no main effects and no interactions.

For Network, there was a very small effect size for “Worktime”, but this was not significant when we look at its *p*-value and confidence interval (see Table 10). This confirms that Worktime only has a very small effect, if it is relevant at all.

**Figure A12:**
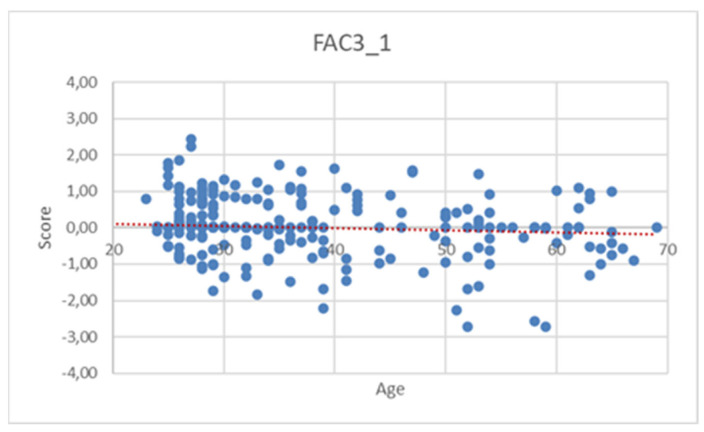
Scatterplot and trendline for PC S3.

For Proximity, there was a very small effect size for “Age”, “Worktime”, and “Role” (see Table 11). Age is very slightly negatively correlated with Proximity; the older the respondent, the slightly less they think proximity contributed to their unplanned meeting (see Figure A12).

**Figure A13:**
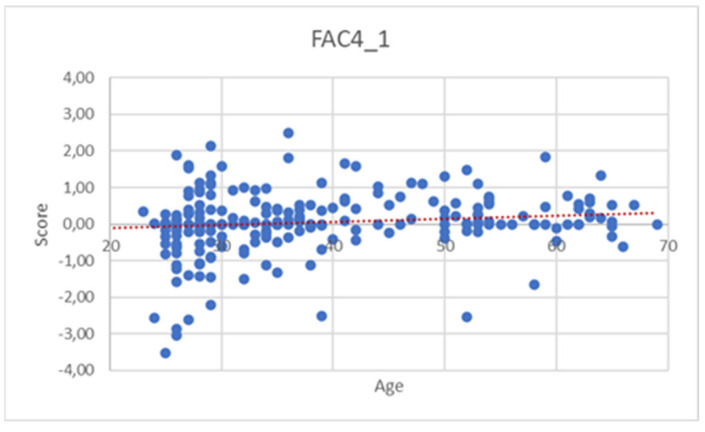
This is a figure. Schemes follow the same formatting.

For Availability, Age is very slightly positively correlated (see Table 12); the older the respondent, the slightly more they feel “Availability” contributed to their interaction (see Figure A13).

**Scheme 1:**
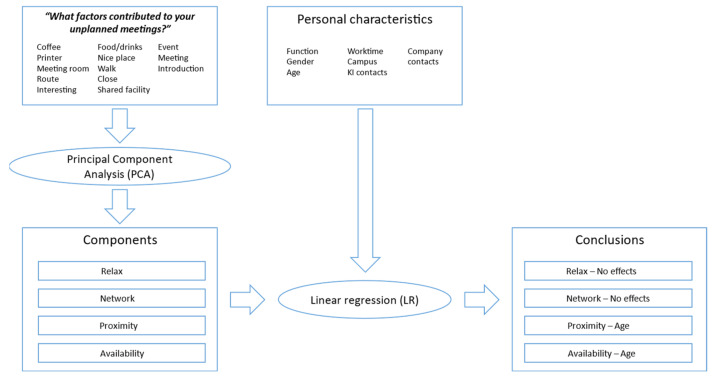
Services—scheme of analysis with principal component analysis (PCA), linear regression analysis and the conclusions.

Scheme 1 summarizes how PCA and LM of the PCs deliver the conclusions described above.

### 3.4. Locations

#### 3.4.1. Respondents

As can be seen in Table 13, 48% of the analyzed responses for locations (N_L_ = 259, see Figure 2) are from RUG, and the majority of respondents are male. There is representation from all levels of academic staff. There is a total of 176 individual respondents (as some gave multiple locations). On average, respondents are 37 years old and have worked at their campus for eight years. They met an average of three people from companies and three people from knowledge institutions in 2019 (see Table 13).

#### 3.4.2. Principal Component Analysis (PCA)

The PCA resulted in three new PCs, explaining 68.11% of the original variance. Table 14 shows the three PCs resulting from this PCA.

The factor loadings in bold in Table 14 make clear that most variables have high loading on only one factor. The last three variables do not load high on any of the three factors. We labeled the three PCs (indicated with a capital letter) and their meaning as follows:

PC1, Aesthetics: Respondents are very positive about their indicated unplanned meeting locations, describing them mostly as pleasant and comfortable, but also attractive, modern, cheerful, colorful, new, beautiful, tasteful and stylish. The indicated locations seem to have in common that they are well designed; this PC was therefore named Aesthetics.

PC2, Cleaned: Respondents indicated that their locations were clean, well-kept and neat. This name has been chosen from the literature, see [39].

PC3, Indoor Environment: The locations were also described as warm (temperature) with good acoustics and quiet. This name has also been chosen from the literature, see [40].

The three variables not included did not contribute to the unplanned meetings or were not relevant enough in distinguishing the specified locations.

#### 3.4.3. Personal Characteristics as Explanatory Variables

As described in the methodology section, a random linear mixed effects regression (LMER) was used to test if personal characteristics such as Age, Worktime, and Role were predictors of the PCs for locations (see Table 15). As explained in Section 2, we used respondents and locations as random factors. In Table 15, we give their variance when they were included as the only variable in the regression analysis, to evaluate their contribution to the differences in PC scores. The text in the interpretation column makes clear that the PC scores are differently related to these two random factors.

To select the relevant personal characteristics, we followed the same procedure as in the linear regression analysis of the services. It is important to note that “Campus” was never selected as a significant variable, indicating that there are no relevant differences between the included campuses.

Aesthetics: The best model included three main effects, although Worktime was not significant, as can be seen in Table 16.

Cleaned: The model with the best AIC was chosen and included Age and Worktime, although Age was not significant, as can be seen in Table 17. The effect of Worktime means that respondents who have worked longer at the university were more positive in their evaluation of the Cleaned component.

Indoor Environment: No effects were found for this PC.

**Scheme 2:**
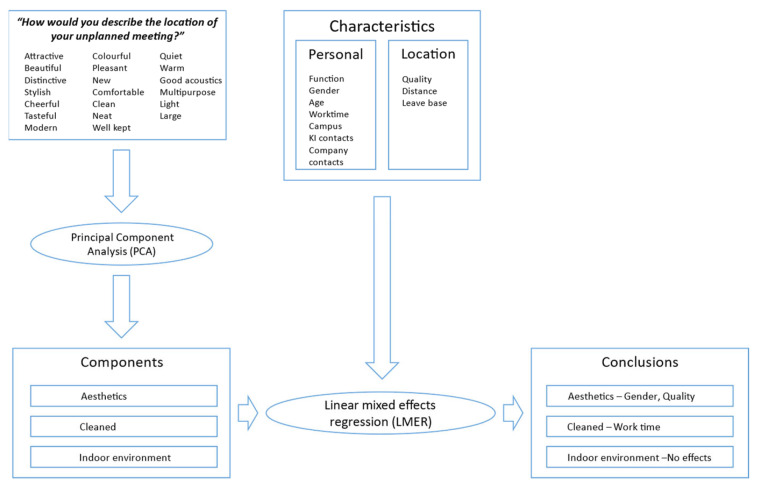
Locations—Scheme of analysis with principal component analysis (PCA) and linear regression analysis (LM).

Scheme 2 summarizes how PCA and the linear mixed regression analysis of the PCs deliver the conclusions described above.

## 4. Discussion

To find the characteristics of successful unplanned or serendipitous meetings and their locations on campus, the survey results were analyzed through spatial descriptives, principal component analysis (PCA), and linear and linear mixed effects regression (LR, LMER). While there is always a risk of respondents not fully completing or misunderstanding the survey, this approach does allow for a large-scale investigation of the campus users’ perspective, providing insight in possible differences between practitioners and users. It was unfortunate that the response from one out of three campuses was significantly lower, but these responses could still be included in the statistical analyses. The number of meetings that was indicated “outside” was low. This could be because respondents did not realize outdoor locations could also be included. It would be recommended in future research to specify this in the survey instructions. The following pattern for contributing services and spatial characteristics was found for successful unplanned meeting locations.

Similarly to the serendipitous communication model described by Peponis, who found that informal interaction nodes such as cafés encourage unplanned meetings [13], the presence of the following services is indicated to strongly contribute to unplanned meetings. It seems these services provide (part of) the necessary affordances as described by Fayard and Weeks [16]. Four principal components (PCs) were identified for services: Relax, Network, Proximity and Availability. Firstly, providing a nice place to be, with good coffee and other food and drink options, that is easy to walk to, contributed to the unplanned meetings. We suggest that a “nice place to be” would be a place that scores highly on the variables mentioned in spatial characteristics (see below). Secondly, events, chance introductions and meetings are also strong contributors. It might therefore be beneficial to have an organizing body on campus to ensure these are part of the campus culture. Thirdly, these services and events should be located close to the general workplace and/or shared facilities that make a location part of a daily route. The maps show that most base locations and most unplanned meeting locations have been indicated in the most densely built part (or center) of the campus. This would therefore seem to be the most suitable location in order to create overall proximity. And fourthly, having spaces, such as a meeting room, available to have a more prolonged conversation once respondents have been introduced, is considered important. Therefore, there should be some flexibility in the campus design or related real-time and online booking system that allows for open/unregularly used locations which provide this space on unplanned occasions.

These four PCs representing evaluative dimensions of campus users can be compared to key factors found in a previous study by these authors, where practitioners—campus directors of 13 Dutch university campuses—indicated what key success factors would facilitate interactions between different campus users on their campus [18]. They identified four categories within a framework of motivators and constraints. These categories were: Designing Spaces, Designing Services, Building Community and Creating Coherence. They indicated key success factors related to food and drink (Relax) and events (Network). Proximity and meeting spaces were also mentioned, but are not separate categories, as they are in this study [18]. The evaluation of campus users therefore seems to be slightly different and puts more emphasis on (immediate) availability of these services.

Some buildings only had indicated locations from respondents with their base location in that same building, while other buildings, such as company buildings or mixed buildings, also had locations indicated by respondents from different buildings (e.g., The Gallery in UT). There also seems to be a traveling connection between buildings with a similar subject matter, such as the Energy Barn and the Energy Academy in RUG. Successful locations score (on average) high on the environmental descriptors by Mehrabian and Russell [19] (Aesthetics) and are Cleaned, with a comfortable Indoor Environment (the three PCs for spatial characteristics).

The pattern of successful locations seems to be that they score well on spatial characteristics (Aesthetics, Cleaned and Indoor Environment), although a higher score does not seem directly related to a higher number of indicated unplanned meetings. To the authors, this suggests that spaces, the “architecture” affordance as mentioned by Fayard and Weeks [16], need to cross a specific threshold of spatial characteristics. When above this threshold, spatial characteristics in themselves seem to no longer add to an increase in unplanned meetings. However, more research is needed on this subject. When comparing these with the key success factors described by campus directors [18], it is interesting to note that Cleaned and Indoor Environment are not mentioned by practitioners, even though these are part of their scope. Perhaps practitioners feel these are too obvious too mention, as they are so integrated in their scope of work, or perhaps these are not considered as much when thinking about the design of a space and not experiencing it in real time when filling in the survey. Either way, it is important for campus managers to realize this impact of the design, but also to keep in mind the connection with the user’s experience. Creating a place that is beautiful, distinctive and stylish could be counterproductive if the design is uncomfortable for the user. The design also needs to include practical implications, such as the ability to be easily kept clean and provide a good indoor environment throughout the year.

These findings suggest that campus users have to be doubly motivated to include opportunities for unplanned meetings in their daily routine. The right services need to be provided, such as lunch and coffee options (Relax), and meetings and events (Network), and they must be close to the users’ daily workplace. Availability even suggests that not only do these services have to be in place, but they also must be (immediately) accessible. To the authors, this suggests that the campus user is a creature of habit. To move outside of their daily routine (which would increase their chances of meeting new people) seems to require an incentive that is both easily accessible, close to their daily activities and interesting/motivational enough to change their daily patterns.

The regression analyses showed that, although some personal characteristics were significant as explanatory variables, all effects were very small. Therefore, these are not very relevant in practice: for example, it seems that the perceptions of unplanned meeting locations and contributing services do not differ between a professor or a PhD student. It is especially interesting to note that no main or interaction effects were found for Campus. In other words, a well-executed campus design may be successful for a wide variety of campus users and locations in The Netherlands. This may greatly help campus directors that want to improve their campus design to further facilitate unplanned meetings. Designs may be reviewed on their merits for the found PCs, the evaluative dimensions, which can help in the decision-making process.

It is interesting to see that the spatial characteristics also include Cleaned and Indoor Environment. A campus’ design should not only take the visuals of the design into account, but also include practicality for easy cleaning and sufficient capacity for installations to ensure a comfortable indoor environment. When looking at Table 15, showing the variance for the random factors in the linear regression, the score on Aesthetics is influenced more by the building, while the score on Cleaned and Indoor Environment is affected more by the respondent. This may mean that, although respondents seem to agree on what Aesthetics are preferable, opinions differ more on what level of Cleaned and Indoor Environment are preferable, which may be hard to include in campus design decision making.

Taking the perspective of the campus user has resulted in a clear framework of evaluative dimensions that trigger behavioral choices. Campus directors may use this framework to reflect on their campus’s design (plans). Comparing these PCs with the key success factors indicated by the campus directors themselves shows many parallels, although this study seems to indicate that the convenience to the user must be emphasized. It seems that even the best and prettiest spaces and services will not motivate users enough to move across the campus, unless it is also convenient. It would be recommended for future research to perform a qualitative study on why this is so important, and to find how this may be integrated in campus designs.

## 5. Conclusions

To find out where on campus unplanned or serendipitous meetings between academic staff and companies take place, and what the characteristics of these places are, this study analyzed a survey of 537 campus users of three different campuses in The Netherlands, using spatial descriptives, principal component analysis and linear regression. The results show that unplanned meetings mostly take place in the most densely built part of the campus, likely because the proximity requirement is most easily fulfilled here. Some respondents indicated meetings within the building where they generally work on campus, while others were visiting other buildings when an unplanned meeting occurred. There seemed to be no relation between people staying inside their building or visiting a different building, and a building’s rating on environmental descriptors.

Through a principal component analysis, firstly, 13 service variables were reduced to four principal components: Relax, Network, Proximity and Availability. The most important variable for each category was: good quality coffee, having a (planned) meeting where you unexpectedly met someone new, in close proximity to your base location and having (a) space available to have a longer conversation once you’ve made a new connection, respectively. Secondly, 20 location variables were reduced to three principal components: Aesthetics, Cleaned and Indoor Environment. The highest scoring variable for each category was: having a pleasant space, a clean space, and a space at a comfortable temperature, respectively. This is in line with the available literature, although campus users seem to put a stronger emphasis on (immediate) availability than campus directors, as found in our previous study [18].

A regression analysis showed that different respondents do not have practically relevant different preferences. Most explanatory variables were not significant; a few were, but these only had very small effects. In addition, a respondent’s campus location was not significant, indicating that this list of principal components can be generally applied to campuses, at least those in The Netherlands.

This indicates that the evaluative principal components mentioned in this paper support the affordances for serendipitous meetings for all campus users across campuses in The Netherlands, when applied to campus design. Such meetings are important in triggering innovative interactions. These evaluative dimensions represent crucial information in designing a university campus and its buildings.

### 5.1. Practical Relevance

These results could be helpful to campus designers and managers as they provide a framework for reviewing a campus’ design and a way to identify possible interventions for an existing campus. The results show that the design should include more than the traditional aesthetics. Food/drink services and events are important motivators for people to visit a location, which should be nearby and available. Once there, a cleaned environment with good indoor environmental quality is important. That means the procurement of services such as cleaning or event organization should not be handled as an afterthought but be included in the campus management of both existing and new buildings.

**Figure 3:**
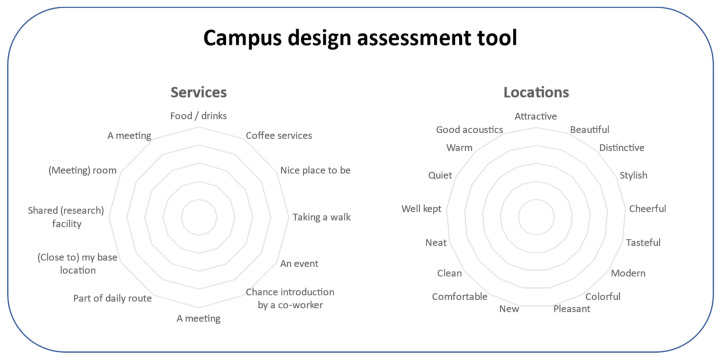
Campus design assessment tool.

To help campus directors implement these findings, a “Campus design assessment tool” was made, see Figure 3. Campus managers can use this tool to conduct a self-assessment, possibly with the help of their advisors, such as architects, or can use the survey method described above to ask their campus users to assess the campus’ current design. The higher an aspect of the design is scored, the further out the corresponding point is placed in the diagram. By connecting the points, a visualization of the campus’ strengths is created.

The study method may also be used to review where current unplanned meetings take place on a campus. Further steps may then be taken to also review why some locations do not have unplanned meetings and how these may be improved. Further research would be recommended applying this method on different campuses in different countries, to find if the same categories apply or if there might be cultural differences.

### 5.2. Strengths and Limitations

By including multiple Dutch campuses in a large-scale survey, this study provides a broad overview of the user’s perspective on what variables can affect the facilitation of unplanned meetings. This allows a comparison with the practitioner’s perspective and a comparison between different campuses. By including both service and location variables, the cohesion of a campus design is preserved, helping to understand the complex relationship between these two from a user’s perspective. Using a quantitative research method, a principal component analysis could be used to find common factors or evaluative dimensions (principal components), creating a practical framework for practitioners. It also allows for regression analysis, providing insight into possible differences in preferences of individual campus users.

Although this paper provides more insight into what evaluative user dimensions can be useful in creating the affordances for unplanned meetings on campuses, some questions require further investigation. Firstly, as this study only included “successful” locations (where unplanned meetings took place), we cannot conclude if the high scores explain (a) the fact they visit this location and not another one, and (b) if these affect respondents’ willingness to interact with strangers, once there. It would be recommended to also include “unsuccessful” locations in future research to see if these questions can be answered. Secondly, this study only included academic staff. It would be recommended for future research to also survey a representative sample of on-campus company employees, to also capture their movement across the campus. Thirdly, the sample of this study may be too small to draw a conclusion on if—and to what extent—there is a traveling connection between buildings with a similar theme. Such a connection is suggested by the data, but a larger study is needed. And fourthly, there does not seem to be a direct relation between a building’s location rating on environmental descriptors and the number of unplanned meetings in that building. This would suggest that a building’s influence is limited: it would need to facilitate unplanned meetings when the opportunity arises, but other things are needed to attract these meetings. To dig deeper into why some buildings attract and others do not, further research is recommended.

## Figures and Tables

**Table 1:** Campus characteristics.

Campus	City	Location	Size (Hectares)	History	Number of Students	Focus
UT	Enschede	Edge of the city	146	Built as a separate entity, with the opportunity for students to live there	12,000	Connecting technology, science and engineering with the social sciences [23]
TUD	Delft	Elongated shape from the city center to the edge of the city and beyond	161	Started as an inner- city university, expanded further away from the city	27,000	Technical university with a mission to create impact for a better society [24]
RUG	Groningen	North of Groningen; on “Campus Groningen”, together with the Hanze University of Applied Sciences	126	Developed in the inner city and later (partially) moved to “ Campus Groningen“	34,000	Aims to connect education and research with sustainable and economic processes through three strategic themes: Energy, Healthy Ageing and Sustainable Society [25]

**Table 2:** Location and service variables.

Location Variables	Service Variables
Pleasant	Coffee services
Attractive	Printer services
Beautiful	(Meeting) room ^1^
Comfortable	Part of my daily route
Cheerful	There is always something interesting going on here
Tasteful	Food/drinks (e.g., lunch)
Good acoustics	It is a nice place to be
Colorful	Taking a walk
Light	(Close to) base location
Neat	Shared (research) facility
Well-kept	An event
Clean	A meeting
Quiet	Chance introduction by a co-worker
Warm (temperature)	
Stylish	
Distinctive	
Large	
Modern	
New	
Multipurpose	

^1^ Having a space (e.g., a room or meeting room) available.

**Table 3:** Personalia variables.

Personalia	Variable Name	Type
Where do you work?	Workplace	Categorical
What is your function/job title?	Function	Categorical
What is your gender?	Gender	Categorical
What is your age?	Age	Continuous
How many years have you been working on the campus?	Worktime	Continuous
How many times have you met people from companies who later became partners in the past calendar year? (2019)	Company contacts	Continuous
How many times have you met people from knowledge institutions who later became partners in the past calendar year (2019)?	KI contacts	Continuous

**Table 4:** Variables assigned by authors.

Assigned by Authors	Variable Name	Type
Has the building been designed with (unplanned) meetings in mind?	Quality	Categorical
What is the respondent’s campus?	Campus	Categorical

**Table 5:** KMO and Bartlett’s test for services.

KMO and Bartlett’s Test—Services	
Kaiser–Meyer–Olkin Measure of Sampling Adequacy	0.747
Bartlett’s Test of Sphericity	
Approx. Chi-Square	742.750
Df	78
Sig.	0.000

**Table 6:** KMO and Bartlett’s test for locations.

KMO and Bartlett’s Test—Locations	
Kaiser-Meyer-Olkin measure of sampling adequacy	0.931
Bartlett’s Test of Sphericity	
Approx. chi-square	4109.432
Df	190
Sig.	0.000

**Table 7:** Respondent characteristics for services.

	Frequency		%	
Campus				
RUG	125		50%	
UT	80		32%	
TUD	44		18%	
Gender				
Female	83		33%	
Male	137		55%	
Other	2		1	
Unknown	27		11%	
Function				
PhD student	79		32%	
Postdoc	30		12%	
Associate/assistant professor	48		19%	
Professor	24		10%	
Researcher	16		6%	
Teacher	12		5%	
Other	21		8%	
Unknown	19		8%	
	Mean	Median	Minimum	Maximum
Age (years)	38	34	23	69
Worktime (years)	7	4	0	35
Meetings with company representatives in 2019	2	0	0	200
Meetings with knowledge institution representatives in 2019	2	1	0	150

**Table 8:** Services, rotated component matrix with four components with their labels and factor loadings on the variables. Factor loading >0.55 are in bold—”Please indicate what variables contributed to your interactions in your indicated locations”.

Rotated Component Matrix ^1^				
	Component			
	RELAX (Component 1)	Network (Component 2)	Proximity (Component 3)	Availability (Component 4)
Food/drinks (e.g., lunch)	**0.82**	0.12	−0.11	0.04
Coffee services	**0.77**	0.06	0.03	0.19
Nice place to be	**0.74**	0.02	0.22	0.24
Taking a walk	**0.60**	0.29	0.29	−0.31
An event	0.16	**0.84**	−0.03	0.12
Chance introduction by a co-worker	0.12	**0.71**	0.21	−0.07
A meeting	0.08	**0.65**	−0.04	**0.56**
Part of my daily route	0.09	0.03	**0.75**	0.12
(Close to) my base location	0.05	−0.02	**0.74**	0.05
Shared (research) facility	0.01	0.45	**0.56**	0.08
(Meeting) room	0.18	0.09	0.23	**0.84**
Printer services	0.39	0.11	0.34	−0.15
There is always something interesting going on here	0.40	0.30	0.42	0.07

^1^ Extraction method: principal component analysis (PCA). Rotation method: varimax with Kaiser normalization. Rotation converged in six iterations.

**Table 9:** Services—Linear regression.

Component	Main Effects	R^2^	*p*	df	Interactions	Interpretation
Relax	-				-	-
Network	Worktime ^1^	0.089	0.039	203	-	Not significant Very small effect size
Proximity	Age	0.067	0.000	224	-	Very small effect size
Availability	Age	0.022	0.003	224	-	Very small effect size

^1^ How many years have you been working on this campus?

**Table 10:** Final regression model for Network.

REGR Factor Score for Network						
Predictors	Estimates	CI		Statistic	*p*	df
(Intercept)	0.134	−0.042–0.310		1.504	1.340 × 10^−1^	223.000
Worktime	−0.015	−0.033–0.002		−1.762	7.948 × 10^−2^	223.000
Observations	225					
R^2^/R^2^ adjusted	0.014/0.009					
AIC	626.411					

**Table 11:** Final regression model for Proximity.

REGR Factor Score Proximity						
Predictors	Estimates	CI		Statistic	*p*	df
(Intercept)	0.776 ***	0.369−1.184		3.754	2.237 × 10^−4^	217.000
Age	−0.019 ***	−0.029–0.009		−3.715	2.586 × 10^−4^	217.000
Observations	219					
R^2^/R^2^ adjusted	0.060/0.055					
AIC	598.947					

*** *p* < 0.001.

**Table 12:** Final regression model for Availability.

REGR Factor Score Availability						
Predictors	Estimates	CI		Statistic	*p*	df
(Intercept)	−0.467 *	−0.882–0.052		−2.217	2.766 × 10^−2^	217.000
Age	0.014 *	0.003–0.024		2.568	1.091 × 10^−2^	217.000
Observations	219					
R^2^/R^2^ adjusted	0.029/0.025					
AIC	606.986					

* *p* < 0.05.

**Table 13:** Respondent characteristics for all locations.

	Frequency		%	
Campus				
RUG	85		48%	
UT	61		35%	
TUD	30		17%	
Gender				
Female	52		30%	
Male	92		52%	
Other	2		1%	
Unknown	30		17%	
Function				
PhD student	39		22%	
Postdoc	16		9%	
Associate/assistant professor	37		21%	
Professor	18		10%	
Researcher	14		8%	
Teacher	8		5%	
Other	19		11%	
Unknown	25		14%	
	Mean	Median	Min	Max
Age (years)	37	36	23	69
Worktime (years)	8	5	1	35
Company meetings in 2019	3	1	1	200
Knowledge institution meetings in 2019	3	1	1	150

**Table 14:** Locations, rotated component matrix with three components with their labels and factor loadings on the variables. Factor loading >0.55 are in bold—“How would you describe this location?”.

Rotated Component Matrix ^1^			
	Component		
	Aesthetics (Component 1)	Cleaned (Component 2)	Indoor Environment (Component 3)
Attractive	**0.84**	0.18	0.29
Beautiful	**0.84**	0.27	0.22
Distinctive	**0.81**	0.20	−0.07
Stylish	**0.79**	0.35	0.16
Cheerful	**0.76**	0.13	0.34
Tasteful	**0.75**	0.30	0.35
Modern	**0.73**	0.49	−0.09
Colorful	**0.70**	0.34	0.19
Pleasant	**0.66**	0.32	0.39
New	**0.65**	0.54	0.00
Comfortable	**0.57**	0.27	0.56
Clean	0.29	**0.83**	0.30
Neat	0.28	**0.79**	0.32
Well-kept	0.42	**0.79**	0.22
Quiet	0.01	0.27	**0.78**
Warm	0.27	0.01	**0.68**
Good acoustics	0.13	0.40	**0.59**
Multipurpose	0.43	0.35	0.31
Light	0.54	0.42	0.24
Large	0.45	0.12	0.22

^1^ Extraction method: principal component analysis (PCA). Rotation method: varimax with Kaiser normalization. Rotation converged in six iterations.

**Table 15:** Locations—The two random factors in the mixed linear regressions.

Component	Variance—Respondent	Variance—Location	Interactions	Interpretation
Aesthetics	0.188	0.381	-	The building accounts for the most variation
Cleaned	0.481	0.240	-	The respondent accounts for the most variation
Indoor Environment	0.574	0.135	-	The respondent accounts for the most variation, even more so than with Cleaned.

**Table 16:** Regression model for Aesthetics.

REGR Factor Score Aesthetics						
Predictors	Estimates	CI		Statistic	*p*	df
(Intercept)	−0.596 ***	−0.944–0.249		−3.362	7.744 × 10^−4^	228.000
Gender [Female]	0.415 *	0.069–0.761		2.348	1.889 × 10^−2^	228.000
Gender [Male]	0.466 **	0.145–0.787		2.848	4.393 × 10^−3^	228.000
Gender [Other]	−0.184	−1.127–0.759		−0.382	7.022 × 10^−1^	228.000
Worktime	0.015	0.000–0.030		1.958	5.020 × 10^−2^	228.000
Quality [1]	0.504 *	0.045–0.962		2.154	3.124 × 10^−2^	228.000
Random Effects						
σ^2^	0.518					
τ_00 respondent_	0.032					
τ_00 building_	0.335					
ICC	0.415					
N_respondent_	173					
N_building_	60					
Observations	237					
Marginal R^2^/Conditional R^2^	0.110/0.479					
AIC	619.644					

* *p* < 0.05, ** *p* < 0.01, *** *p* < 0.001.

**Table 17:** Regression model for Cleaned.

REGR Factor Score Cleaned						
Predictors	Estimates	CI		Statistic	*p*	df
(Intercept)	−0.142	−0.471–0.187		−0.848	3.967 × 10^−1^	231.000
Age	−0.000	−0.009–0.008		−0.016	9.872 × 10^−1^	231.000
Worktime	0.020 *	0.001–0.040		2.030	4.237 × 10^−2^	231.000
Random Effects						
σ^2^	0.481					
τ_00 respondent_	0.097					
τ_00 building_	0.369					
ICC	0.492					
N _respondent_	173					
N _building_	60					
Observations	237					
Marginal R^2^/Conditional R^2^	0.027/0.506					
AIC	631.903					

* *p* < 0.05.

**Figure A11:**
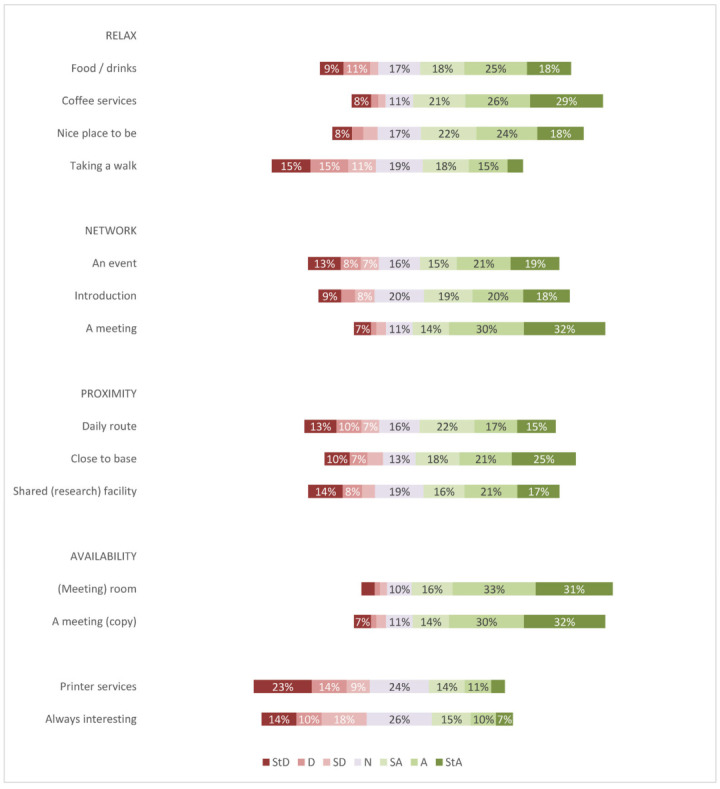
Likert scale 1–7 scores for services. StD = strongly disagree, D = disagree, SD = slightly disagree, N = neutral, SA = slightly agree, A = agree, StA = strongly agree.

**Figure A14:**
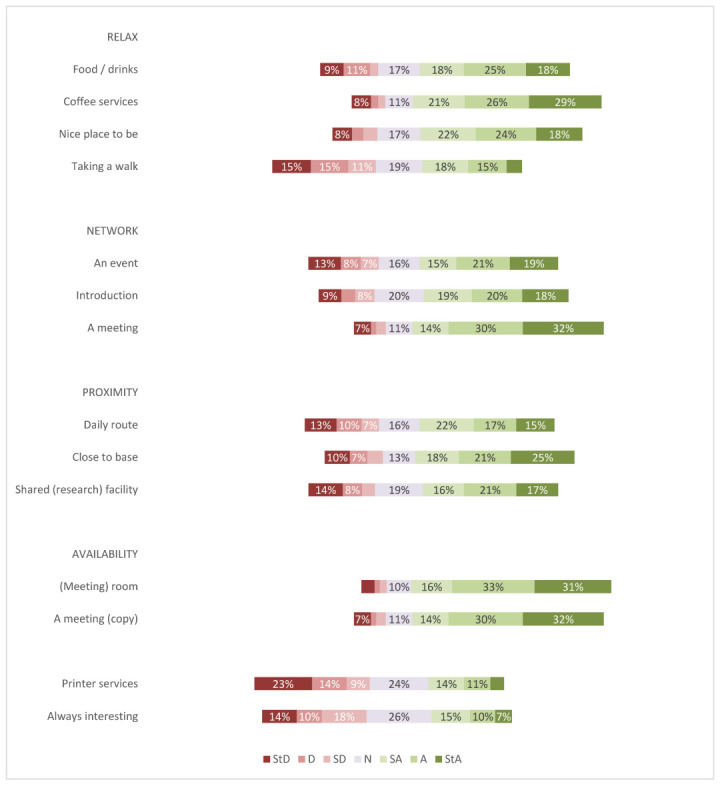
Locations—overview Likert scores. StD = strongly disagree, D = disagree, SD = slightly disagree, N = neutral, SA = slightly agree, A = agree, StA = strongly agree.

## Data Availability

Not applicable.

## Appendix A

**Table A1:** Respondent characteristics for company locations.

Characteristic	N	%	
Campus			
RUG	50	45%	
UT	39	35%	
TUD	23	21%	
Gender			
Female	25	22%	
Male	64	57%	
Other	2	2%	
Unknown	21	19%	
Function			
PhD	24	21%	
Postdoc	8	7%	
Associate/assistant professor	23	21%	
Professor	15	13%	
Researcher	8	7%	
Teacher	5	4%	
Other	12	11%	
Unknown	17	15%	
Characteristics	Average	Min	Max
Age	39	3	69
Worktime	9	1	35
2019 companies	3	1	25
2019 knowledge institutions	3	1	8

**Table A2:** Respondent characteristics for knowledge institution locations.

Characteristic	N	%	
Campus			
RUG	62	51%	
UT	39	32%	
TUD	21	17%	
Gender			
Female	40	33%	
Male	68	56%	
Other	1	1%	
Unknown	13	11%	
Function			
PhD	25	20%	
Postdoc	13	11%	
Associate/assistant professor	29	24%	
Professor	12	10%	
Researcher	10	8%	
Teacher	5	4%	
Other	17	14%	
Unknown	11	9%	
Characteristics	Average	Min	Max
Age	37	3	69
Worktime	8	1	35
2019 companies	3	1	25
2019 knowledge institutions	3	1	15
